# Fatal Adverse Events of Dabigatran Combined With Aspirin in Elderly Patients: An Analysis Using Data From VigiBase

**DOI:** 10.3389/fphar.2021.769251

**Published:** 2021-12-22

**Authors:** Qingxia Zhang, Qian Ding, Suying Yan, Qun-Ying Yue

**Affiliations:** ^1^ Department of Pharmacy, Xuanwu Hospital Capital Medical University, National Clinical Research Center for Geriatric Disease, Beijing, China; ^2^ School of Pharmaceutical Science, Capital Medical University, Beijing, China; ^3^ Uppsala Monitoring Centre, Uppsala, Sweden

**Keywords:** dabigatran, aspirin, elderly, fatal, adverse drug events, VigiBase

## Abstract

**Introduction:** The elderly are vulnerable to cardiovascular diseases and the incidence of atrial fibrillation (AF) and venous thromboembolism (VTE) increases significantly with age. Dabigatran is a commonly used new oral anticoagulant approved by the FDA for stroke prevention in patients with non-valvular AF and VTE treatment and prevention. Aspirin is commonly used as a preventive drug for cardiovascular diseases. AF and coronary heart disease share many risk factors, so these two diseases often coexist and thus dabigatran and aspirin are often combined in those people. The aim of this study was to analyze the clinical characteristics of fatal adverse events of dabigatran combined with aspirin in elderly patients, and to provide references for clinical rational use of drugs.

**Materials and Methods:** Fatal adverse events related to the combined use of dabigatran and aspirin in elderly patients aged over 75 were extracted from the WHO global database of individual case safety reports (VigiBase). Well-documented reports, vigiGrade completeness score ≥0.80, or with an informative narrative, were analyzed with a focus on the clinical features of the cases.

**Results:** From 1968 up to January 19, 2020, there were 112 eligible reports in VigiBase from 13 countries, of which 33 were identified as well-documented. Of these 33, 19 were male (58%) and 14 were female (42%), the average age of the patients was 84 (75–95 years), with five cases of extreme weights (>100 kg in one case, <50 kg in four cases). There were 31 cases of death by internal bleeding (mainly 15 of gastrointestinal hemorrhage and 12 of intracranial hemorrhage) and two cases of the sudden death of unknown cause. Medication errors existed in 15 patients. The times to onset (TTO) was provided in 24 cases, ranging from 2 days to 4 years, and in 12 patients occurred within a month. Of the 31 patients with fatal bleeding events, 29 were associated with other factors that increase the risk of bleeding, such as diseases (hypertension, renal impairment, stroke, gastrointestinal related diseases, hypothyroidism, and cancer), drugs (antiplatelets, anticoagulants, thrombolytics, P glycoprotein substrates, non-steroidal anti-inflammatory drugs, hormones, selective serotonin reuptake inhibitors, and acetaminophen) and other factors (low body weights and alcohol consumption), and 21 of these contained two or more risk factors.

**Conclusion:** The fatal adverse events associated with the combined use of dabigatran and aspirin in elderly patients were mainly serious bleeding events, which often occurred within 1 month. Most of these cases had medication errors and most of the patients had multiple diseases, medications, or other conditions at the same time that increase the risk of bleeding. It is suggested that prescription of dabigatran and aspirin in elderly patients should go along with alertness for medication errors, care for correct dose or control of other bleeding risk factors, and the combined medication time should be as short as possible to minimise serious adverse events.

## Introduction

The elderly are vulnerable to cardiovascular diseases. Among patients with ST-segment elevation myocardial infarction (STEMI), people aged ≥75 years accounted for 18%, and among patients with non-ST-segment elevation myocardial infarction (NSTEMI), people aged ≥75 years accounted for 25% (Amsterdam et al., 2014). Aspirin is commonly used as a preventive drug for cardiovascular diseases.

The incidence of atrial fibrillation (AF) and venous thromboembolism (VTE) increases significantly with age. The incidence of AF gradually increases every 10 years of life (Wolff et al., 2015), and the elderly account for 45% of the AF population (Go et al., 2001). The risk of VTE in patients over 40 approximately doubles every 10 years (Heit et al., 2016). In addition, the risk of stroke increases significantly with age, so preventing stroke is particularly important for elderly patients with AF (Wolf et al., 1991). Compared to warfarin, the new oral anticoagulants (NOACs) reduce more ischemic stroke and bleeding events in elderly patients ≥75 years (Ruff et al., 2014). Therefore, NOACs are recommended as the first choice of anticoagulant therapy in patients 75 years and over with AF (Hindricks et al., 2021). Dabigatran is a commonly used NOAC approved by the FDA for stroke prevention in patients with non-valvular atrial fibrillation and VTE treatment and prevention.

AF and coronary heart disease share many risk factors, so these two diseases often coexist. The incidence of AF in patients with coronary heart disease is 6–21% (Schmitt et al., 2009), while the incidence of coronary heart disease in patients with AF is 20–30% (Nieuwlaat et al., 2005; Nabauer et al., 2009; Kralev et al., 2011), so dabigatran and aspirin are often combined in elderly patients.

In order to understand the potential reasons for death in elderly patients treated with dabigatran and concomitant aspirin, we performed this study aimed to analyze the clinical characteristics of fatal cases in above-mentioned patients from the WHO global database of individual case safety reports (VigiBase) and provide references for clinical rational drug use. In order to better describe patient characteristics, we decided to focus this analysis exclusively on well documented cases.

## Materials and Methods

### Data Source

The data of this study were derived from VigiBase from 1968 to January 19, 2020. The active ingredients of the drugs were acetylsalicylic acid (aspirin) and dabigatran, which were regarded as suspected/interacting drugs. Elderly patients are defined as ≥75 years referring to the AHA/ACC guideline (Amsterdam et al., 2014). All fatal adverse event reports were extracted.

### Data Extraction

Adverse events were classified using the System Organ Classification (SOC) and Preferred Term (PT) of the international Medical Dictionary for Regulatory Activities (MedDRA). All the useful variables contained in the reports were considered, including 1) case information: the category of reporters and the country of cases; 2) patient information: age, gender, weights, relevant medical history and drinking history; 3) medication information: suspected and concomitant medication indications, dosage, and medication time; 4) description of adverse events: clinical manifestations and the times to onset (TTO), the tests and procedures performed, treatment and outcome. The criteria for well-documented cases (Bergvall et al., 2014) was vigiGrade completeness score ≥0.80 or with informative narratives (such as allowing causality assessment and analysis of the clinical characteristics of the cases).

## Results

As of January 19, 2020, vigibase had received 112 fatal adverse event reports of dabigatran combined with aspirin in elderly patients aged over 75 years, involving 21 SOCs, from 13 countries, the top three being the United States (55 cases, 49%), Japan (17 cases, 15%) and France (15 cases, 13%). The highest proportion for reporter qualification was doctors (51 cases, 46%), followed by consumers or non-health professionals (27, 24%), pharmacists (19, 17%), and other health professionals (18, 16%). Among 112 patients, 66 were male (59%) and 45 were female (40%). The average age was 83 (range 75–97 years), of which 71 patients (63%) were 80 or over. In addition to dabigatran and aspirin, the main drugs listed as suspected/interactions were: antiplatelet drugs (27 cases), anticoagulants (20), non-steroidal anti-inflammatory drugs (NSAIDs, four) and other drugs (four cases of propafenone, two of sotalol, three of tramadol and two of oxycodone). Among the 112 cases, there were 103 bleeding events, most occurring in the gastrointestinal system (51 cases) and nervous system (36), and 13 non-bleeding events, which mainly manifested as renal failure (9 cases) and coronary artery embolism (3).

Among the 112 reports, 33 (29%) were classed as well-documented. The top three were France (ten, 30%), Japan (6, 18%) and Germany (four, 12%) among the 11 countries. The reporters were doctors (28 cases, 85%, plus two co-reported by doctors and pharmacists or other health professional), pharmacists (five cases, 15%), and other health professional (two, 6.1%). Among the 33 patients, 19 were male (58%) and 14 were female (42%). The average age was 84 (range 75–95 years), of which 25 patients (76%) were 80 or over. There were five patients with extreme weights, including one with obesity (120 kg) and four low weights (<50 kg). The fatal adverse events were sudden death of unknown cause in two cases (6.1%) and hemorrhage in 31 cases, including gastrointestinal hemorrhage in 15 (46%), intracranial hemorrhage in 12 (36%), hemothorax in two (6.1%), pulmonary hemorrhage in one (3.0%), and renal hematoma in one (3.0%). Among the 15 patients reported as gastrointestinal bleedings: upper gastrointestinal bleeding in four cases, lower gastrointestinal bleeding in five, bleeding location not provided in six cases. No one had taken proton pump inhibitor (PPI) in the four upper gastrointestinal bleeding patients, but PPI was taken by three of five patients with lower gastrointestinal bleeding and three of six where bleeding locations not provided.

In 15 patients medication errors were noted, including two cases of contraindications (severe renal impairment, creatinin clearance <30 ml/min), two cases of overdoses (“dabigatran, 150 mg, twice daily” when combining amiodarone and “acetaminophen, 1,000 mg, twice daily”), 11 of over treatment (ten of excess antiplatelet therapy: three of AF patients, five of AF with ischemic stroke patients, one of AF with stable coronary heart disease without high risk of ischemia and one of AF with post-percutaneous coronary intervention (PCI) for 2 years; one of the unnecessary duration: AF with post-PCI dual antiplatelets therapy more than 2 months). Five patients had a history of previous PCI and none of coronary artery bypass grafting (CABG), of which two patients had information on the PCI surgery time: one was in the last 12 months and the other one was longer than 12 months. A totol of seven patients had a history of stroke. Contraindications and overdoses are bolded while overtreatments are shown in italic in Table 1. In 24 reports there was information on TTO, ranging from 2 days to 4 years, and in 12 patients the events occurred within a month.

**TABLE 1:** Characteristics of the well-documented fatal cases of dabigatran combined with aspirin in elderly patients.

case	Age (years)	Gender	Dabigatran dosage	Aspirin dosage	TTO	Main clinical manifestations	Main co-reported medications	Relevant conditions
*1*	89	M	110 mg bid	*75 mg qd*	10d	Hematemesis Melena	*Clopidogrel 75 mg qd, Enoxaparin 4000IU qd,* acetaminophen	AF, Post-PCI, Post-THR, HT, Rectal-sigmoid colon adenocarcinoma
*2*	79	M	110 mg q12h	*100 mg qd*	1y	Upper gastrointestinal hemorrhage		AF, Gastric angiogenesis
**3**	76	M	**150 mg bid**	75 mg qd	2m	Gastrointestinal hemorrhage	Amiodarone	AF, Hypothyroidism, CKD
4	89	F	110 mg bid	75 mg qd	2d	Sudden death	Acetaminophen 75 mg qd, Amiodarone	AF, Ischemic cardiomyopathy, HT
5	80	M	110 mg bid	75 mg qd	10d	Cardio-respiratory arrest	Amiodarone	Post-TKR,HT, Obesity (120 kg)
6	85	M	Unknown	Unknown	1m	Lower gastrointestinal hemorrhage		AF, Ischemic cardiomyopathy, HT
7	87	M	110 mg bid	100 mg qd	3d	Gastrointestinal hemorrhage		AF, HT, Colon cancer, Prostate cancer
8	85	M	110 mg bid	100 mg qd	2m	Hemothorax	Beraprost 40ug tid	HT, Alcohol, CKD
9	75	F	110 mg q12h	150 mg Unknown	Unknown	Renal hematoma	Clopidogrel 75 mg	Post-PCI, NSTEMI
10	81	F	75 mg bid	100 mg qd	2d	Intracranial hemorrhage	Verapamil 40 mg tid	AF, Angina pectoris, HT, CKD, Low weight (38.5 kg)
*11*	80	M	110 mg bid	*100 mg qd*	1y5m	Intracranial hemorrhage		AF, Stroke
12	79	M	75 mg bid	75 mg qd	Unknown	Intracranial hemorrhage	Alteplase	Unknown
**13**	78	M	110 mg bid	Unknown	2d	Pulmonary hemorrhage	Ticagrelor 180 mg qd, heparin	AF, NSTEMI, **Renal Failure**
*14*	85	M	110 mg bid	*75 mg qd*	12m	Intraventricular hemorrhage		Arrhythmia
15	87	F	110 mg bid	100 mg qd	4y	Ulcer hemorrhage	Rivaroxaban 10 mg qd	AF, Middle artery dissection, gastroduodenal ulcer; Low weight (43 kg)
16	85	F	110 mg q12h	100 mg q24h	3m	Gastrointestinal hemorrhage		AF
*17*	87	F	110 mg bid	*75 mg qd*	1m	Intracranial hemorrhage		Arrhythmia, Myocardial infarction, Stroke
*18*	80	F	110 mg Unknown	*300 mg qd*	3d	Intracranial hemorrhage	Dalteparin sodium 500DF, alteplase	AF, Stroke
**19**	90	M	110 mg bid	100 mg qd	16d	Gastrointestinal hemorrhage		**Renal failure**, HT; Low weight (39 kg)
*20*	77	M	110 mg bid	*100 mg qd*	10d	Intracranial hemorrhage		AF, Stroke, HT, Prostate cancer
21	95	F	110 mg bid	100 mg Unknown	Unknown	Intestinal hemorrhage	Acetaminophen 325mg, Diclofenac	AF, Ischemic cardiomyopathy, HT, CKD
*22*	86	F	110 mg bid	*80 mg qd*	2m	Intracranial hemorrhage		AF, Hypothyroidism, Low weight (41 kg)
*23*	84	M	110 mg bid	*Unknown*	6m	Intracranial hemorrhage	Dipyridamer 75 mg bid, Sertraline	AF, HT, CKD, Gastritis, Stroke
**24**	93	F	110 mg bid	75 mg qd	3d	Gastrointestinal hemorrhage	**Acetaminophen 1,000** **mg bid**	AF, ACS, HT, Gastrointestinal bleeding
25	80	F	110 mg bid	100 mg qd	3d	Rectal hemorrhage	Clopidogrel 75 mg qd	Unknown
26	75	M	150 mg Unknown	Unknown	Unknown	Intracranial hemorrhage	Escitalopram	AF, Post-PCI, HT
*27*	88	F	110 mg bid	*Unknown*	3m	Intracranial hemorrhage		AF
28	76	M	Unknown	Unknown	9m	Rectal hemorrhage	Prednisone, Analgin, Omeprazole	AF, Dilated cardiomyopathy, HT
29	88	F	110 mg bid	100 mg qd	Unknown	Rectal hemorrhage	Analgin, Omeprazole	AF, Post-PCI, stroke
30	86	M	110 mg bid	Unknown	Unknown	Pulmonary alveolar hemorrhage; Rectal hemorrhage	Enoxaparin 0.4 ml qd, heparin 12500IU tid	AF
31	89	F	110 mg bid	100 mg qd	Unknown	Hemothorax		AF, HT
*32*	89	M	110 mg bid	*100 mg qd*	Unknown	Upper gastrointestinal hemorrhage		AF, Post-PCI, HT, CKD, Amiodarone-related hypothyroidism, stoke
33	86	M	Unknown	100 mg qd	Unknown	Intracranial hemorrhage	Clopidogrel 75 mg qd	AF, HT

TTO: the times to onset; M:male; F:female; AF: atrial fibrillation; PCI: percutaneous coronary intervention; THR: total hip replacement; TKR: total knee replacement; VTE: venous thromboembolism; DVT: deep vein thrombosis; NSTEMI: non-ST-segment elevation myocardial infarction; ACS: acute coronary syndrome; HT: hypertension; CKD: chronic kidney disease. The meaning of the bold values is “contraindications and overdoses” and italic means overtreatments.

Among the 31 fatal cases with bleeding events, 29 had other diseases, drugs, or conditions that increase risk of bleeding. The concomitant diseases included hypertension in 17 cases, renal impairment in eight, stroke in seven, gastrointestinal diseases in four, hypothyroidism in three, and cancer in three cases. The concomitant medications that increased the risk of bleeding were antiplatelet drugs in seven cases (four of clopidogrel, one of dipyridamole, one of ticagrelor, one of beraprost), five of anticoagulant drugs (three of low molecular weight heparin, one of heparin, and one of rivaroxaban), five of P-glycoprotein (P-gp) substrate (three of amiodarone, one of verapamil, and one of levetiracetam), four of acetaminophen, one of NSAIDs (diclofenac), two of non-addictive analgesic (metamizole), two of thrombolytic drugs (alteplase), two of selective serotonin reuptake inhibitor drugs (SSRIs, one of sertraline and one of escitalopram), and one of corticosteroid (prednisone). Other conditions were low weights in four cases (<50 kg) and alcoholism in one case. Twenty-one patients had two or more risk factors for bleeding, and at most six of the above risk factors were concurrent. The clinical examinations performed for the bleeding events mainly included gastroscopy, colonoscopy, brain MRI, and CT angiography. Laboratory tests mainly included blood routine, renal function, blood coagulation function, and blood concentration determination of dabigatran. Withdrawal of the suspected drugs was the primary treatment method, and two patients were treated with idarucizumab, a dabigatran specific antagonist.

## Discussion

The fatal adverse events relating to the combined use of dabigatran and aspirin in elderly patients mainly manifested as severe bleeding. The 2021 European Heart Rhythm Association Practical Guide recommended the HAS-BLED score (Pisters et al., 2010) [Hypertension (uncontrolled, >160 mmHg systolic), Abnormal renal and liver function, Stroke, Bleeding history or predisposition, Labile INRs, Elderly (>65 years), Drugs (antiplatelet agents or NSAIDs) or alcohol concomitantly] (Pisters et al., 2010) to assess the bleeding risk of anticoagulants in AF patients (Steffel et al., 2021). However, previous studies have shown that the HAS-BLED score may underestimate the bleeding risk (Caldeira et al., 2014; Senoo et al., 2016), which was also suggested by our research with the findings that some risk factors that not mentioned in HAS-BLED (like hypothyroidism, cancer, combining P-gp substrate or hormones or SSRIs or acetaminophen, and low body weights) need to be considered. In addition to the risk factors for bleeding in the focus of this study (elderly, and the concomitant use with aspirin), the subjects were mostly exposed to other factors increasing bleeding risk, including other diseases, medications, and conditions which are discussed below.

Hypertension is one of the risk factors for AF (Huxley et al., 2011). The guidelines (Steffel et al., 2021) recommended the CHA2DS2-VASc score (Lip et al., 2010) (Congestive heart failure/left ventricular dysfunction, Hypertension, Age≥75, Diabetes mellitus, Stroke/TIA/TE, Vascular disease, Age 65–74, Sex category) to assessthe risk of stroke in patients with AF, which means hypertension is also one of the main risk factors for stroke. Meanwhile, uncontrolled hypertension (>160 mmHg systolic) increases the risk of intracranial hemorrhage (Rodriguez-Luna et al., 2013), which is also listed by the HAS-BLED score (Pisters et al., 2010). Therefore, it is necessary for patients with AF and hypertension to actively control their systolic blood pressure below 160 mmHg.

In patients with chronic renal insufficiency (CKD), the clearance rate of dabigatran may decrease and the risk of bleeding may increase. Patients on dabigatran should therefore adjust the dose according to renal function (Steffel et al., 2021). Naccarelli and co-workers (Naccarelli et al., 2020) pointed out that drug interaction might lead to improper dosage or contraindication of anticoagulants in patients with CKD, but there was a lack of analysis in previous studies. In this study, six of eight elderly CKD patients were treated with drugs with a known risk of interaction, including NSAIDs and P-gp inhibitors, which potentially caused over-dosing. Kumar and co-workers (Kumar et al., 2019) showed that the well-known bleeding score has not been verified in CKD patients and cannot be used for clinical decision-making alone. Therefore, it is recommended that the renal function of the elderly patient should be evaluated before starting anticoagulants. For patients with CKD, dabigatran should be prescribed with care, and the medication list should be reviewed regularly to avoid improper combinations. It is advised to conduct renal function assessments and bleeding risk screening frequently and reduce the dosage when necessary.

Hypothyroidism may also decrease the clearance of dabigatran. In our study, two of three hypothyroidism patients also had renal insufficiency, which would further increase the risk of decreasing the clearance, and there were similar cases in previous reports (Kernan et al., 2012). Attention should be paid to monitoring the coagulation function and adjusting the dose in time when thyroid function declines during anticoagulation therapy.

Two patients suffered from tumors of the digestive tract system, and major gastrointestinal bleeding occurred after anticoagulant therapy. At present, there is controversy about the risk of bleeding during anticoagulant therapy in cancer patients. Previous study (Schulman et al., 2016) has suggested that the management of bleeding in cancer patients taking NOACs does not pose a greater challenge than non-cancer patients. However, Koike (Koike et al., 2018) discussed that, compared with patients without cancer, AF patients with cancer had a higher incidence of bleeding when receiving dabigatran, and major bleeding in cancer patients on dabigatran therapy was mainly gastric, due to gastrointestinal cancer. Flack (Flack et al., 2017) recommended gastrointestinal cancer screening before or during anticoagulation therapy. We recommend that attention should be paid to the possibility of increased bleeding risk when prescribing anticoagulation therapy for cancer patients, especially gastrointestinal cancer, and they should be checked more frequently for hidden bleeding when other risk factors for bleeding are present.

The Institute for Safe Medication Practices considers NOACs as high-alert drugs because patients are at higher risk of major harm when they are used incorrectly. In this study, medication errors accounted for a relatively high proportion (15/33), especially overtreatment. The elderly often have multiple diseases and are subject to polypharmacy, which is likely to increase the risk of adverse reactions such as bleeding, while over-treatment will further increase the risk. AF patients without indications for antiplatelet therapy, including patients with ischemic stroke and patients with stable coronary heart disease without high ischemic risk, should avoid combined antiplatelet therapy. Patients with elective PCI may benefit from dual antithrombotic therapy, however the short duration of antiplatelet therapy is recommended. The guidelines (Hindricks et al., 2021; Steffel et al., 2021) recommend anticoagulation combining dual antiplatelet drugs therapy for one to 7 days (maximum 1 month), then NOACs combined with P_2_Y_12_ inhibitors for 6 months (maximum 12 months), before switching to mono-NOAC. Dose adjustment is necessary for dabigatran according to creatinine clearance, age, and co-medications. Dabigatran should be contraindicated for severe renal insufficiency. Amiodarone is a P-gp inhibitor, which increases by 12–60% the concentration of dabigatran. Low-dose dabigatran (110 mg, bid) is required if there is a high risk of bleeding (Steffel et al., 2021). In our study, the patient in case 3 (Table 1) was elderly, with CKD, and taking aspirin at the same time, and thus had a high risk of bleeding. The standard “150 mg, twice a day” is excessive and should be reduced. Dabigatran is a P-gp substrate, so the concomitant use of P-gp inhibitors may increase the blood concentration of it and cause bleeding (Steffel et al., 2021). A study showed that when dabigatran is used in combination with common antihypertensive drugs such as verapamil and diltiazem and other moderate to strong P-gp inhibitors, caution is required regardless of the patient’s renal function. Related studies have also shown that the degree of interaction between dabigatran and verapamil depends on the time of administration. When verapamil was given 2 hours after dabigatran, the Cmax and AUC of dabigatran only increased by 20 and 10%, respectively, but when given 1 hour before dabigatran, the Cmax and AUC of dabigatran may be increased by 180 and 150% (Wiggins et al., 2020). Considering that elderly patients who need anticoagulation combined with antiplatelet therapy often have multiple risk factors for bleeding, we recommend that clinicians prioritize NOACs other than dabigatran when given with moderate-strength P-gp inhibitors. If dabigatran must be used, consider drugs that do not cause interaction. If the patient has to use these two drugs at the same time, it is recommended to take verapamil 2 h after dabigatran. For drugs that increase the risk of ulcers, such as NSAIDs and corticosteroids, they should also be avoided as much as possible in elderly patients who need anticoagulation therapy to minimise major gastrointestinal bleeding. Studies have shown that when oral anticoagulants or antiplatelet drugs are concomitantly used with SSRIs, the risk of intracranial hemorrhage or gastrointestinal bleeding is increased (Hackam and Mrkobrada, 2012; Jiang et al., 2015). Overdosing of acetaminophen can cause severe liver damage (Raffa et al., 2014), which can lead to coagulation dysfunction and increase the risk of bleeding in patients undergoing antithrombotic therapy.

The dosage of dabigatran is more susceptible to be influenced by bodyweight among all NOACs (Steffel et al., 2018). Low body mass index may be an independent predictor of dabigatran bleeding events. When the body weight is less than 50 kg, a dabigatran dose of “110 mg, bid" (Steffel et al., 2018) is recommended, and the trough blood concentration should be monitored as necessary (Steffel et al., 2021). Elderly female patients with low body weight will present decreased creatinine clearance rate, so more attention should be paid.

Fatal hemorrhage mainly occurred in the intracranial and gastrointestinal systems. The position paper of the Italian National Association of Hospital Cardiologists (ANMCO) and the Italian Association of Hospital Gastroenterologists and Endoscopists (AIGO) argued that the concomitant intake of anticoagulants and antiplatelet drugs increased upper and lower gastrointestinal bleeding risk by 60 and 30% respectively (Abrignani et al., 2021). Therefore, the management of gastrointestinal bleeding is very important for patients on dual antithrombotic therapy. Research by ANMCO and AIGO showed that PPI not only helped prevent low-dose aspirin-induced ulcers, but also reduced the occurrence of anticoagulation-related bleeding adverse events (Abrignani et al., 2021). However, a study has shown that PPI are ineffective in preventing bleeding in the small intestine or colon (Scarpignato et al., 2016). This may be the reason for severe rectal bleeding after the combined use of PPI in cases 28 and 29 (Table 1). Therefore, for patients on dual antithrombotic therapy, the use of PPI is not a perfect protective factor, and regular follow-up, evaluation and monitoring are still important preventive measures. Patients with intracranial hemorrhage may fall through dizziness and headache. They may also fall due to accidents or other risk factors and cause severe intracranial hemorrhage because of anticoagulant and antiplatelet effects. Therefore, attention should be paid to remind patients to be alert for falls, and to screen risk factors, such as the combination of multiple chronic diseases, cognitive impairment, alcoholism, and polypharmacy. Studies have shown that the use of amiodarone may increase the likelihood of falls in elderly patients with AF by postural hypotension, embolic events, side effects, and/or other heart rhythm disturbances, and the risk was highest within the first 14 days (Santos et al., 2012; Dalgaard et al., 2019). For patients undergoing antithrombotic therapy, the risk of fatal bleeding after falling is significantly increased. Therefore, clinical attention should be paid to comorbidities and concomitant medications that may increase the risk of falls in elderly patients with AF, particularly when prescribing amiodarone.

Twelve (12/24, 50%) elderly patients had fatal bleeding adverse events within 1 month of the combination of dabigatran and aspirin. It is therefore suggested to monitor such events, in particular during the first month after the combination medication, and thereafter the risk factors of patients should be monitored dynamically, and timely intervention should be made when the risk of bleeding increases.

Some patients will have relevant symptoms or signs before the irreversible severe bleeding events. Blood in stool and other gastrointestinal bleeding symptoms are easy for the patient to notice, while bleeding symptoms in other systems may be likely overlooked. For example, patients with alveolar hemorrhage have had progressive difficulty breathing, and patients with intracranial hemorrhage may experience dizziness and headaches. Patients should be reminded to pay attention to the early symptoms of bleeding and thrombosis, and seek medical treatment as soon as possible as appropriate to prevent further serious events. Some auxiliary software such as the VerifyNow system is widely used in patients on antiplatelet therapy as an attempt to predict post-operative haemorrhage and is recommended in a consensus document as a tool for clinicians for predicting thrombotic and bleeding events in patients undergoing DAPT (Aradi et al., 2014; Cimmino et al., 2020).

The reversal effect of idarucizumab on dabigatran has been confirmed (Pollack et al., 2015). For patients with severe bleeding caused by dabigatran, it is recommended to use idarucizumab immediately for reversal and monitor the vital signs continuously. Percutaneous Left Atrial Appendage Occlusion (LAAO) represents a safe alternative for those patients with contraindications for long-term oral anticoagulation and high bleeding risk or after experiencing life-threatening bleedings (Cimmino et al., 2021).

Due to the nature of spontaneous reporting, a limitation of this study is that there was a lack of information about important clinical parameters and concomitant diseases or other conditions in most of the cases (about 70%). Hence, we have selected and analyzed the well-documented reports. In addition, the risk of particularly devastating consequences deriving from concomitant antithrombotic drugs used post-marketing could be higher than reported in controlled clinical trial as underlined in several previous studies (Gulati et al., 2018; Menditto and Antonicelli, 2020; Said et al., 2020). However, the magnitude of this risk cannot be determined in this study due to the nature of spontaneous reporting. Therefore, instead of quantitative assessments, for example, using the HAS-BLED score, we can only qualitatively assess the risk factors for fatal adverse events in the study population based on the data from VigiBase.

## Conclusion

The fatal adverse events in elderly patients who concomitantly used dabigatran and aspirin were mainly gastrointestinal and intracranial hemorrhage. In most of the cases co-morbidities and other concomitant drugs may have aggravated the bleeding. It is particularly important to evaluate bleeding risk factors for elderly patients before combining dabigatran and aspirin, and throughout treatment. During the treatment, conditions that may aggravate bleeding should be actively corrected, concomitant drugs that may increase bleeding risks should be used rationally with caution or be avoided as appropriate, and gastrointestinal and intracranial bleeding events should be closely monitored. At the same time, it is necessary to inform physicians and patients on the importance to be alert to risk factors for bleeding adverse events, such as avoiding falls, controlling blood pressure, adopting a healthy lifestyle and correct medication methods, and be aware of possible early bleeding signs and symptoms. The patients should seek medical attention as soon as possible if bleeding does not stop. For patients who are at risk of fatal bleeding, it is recommended to initiate idarucizumab for immediate rescue.

## Data Availability

The original contributions presented in the study are included in the article/Supplementary Material, further inquiries can be directed to the corresponding authors.
